# Draft Genome Sequence of *Acidovorax* sp. Strain NB1, Isolated from a Nitrite-Oxidizing Enrichment Culture

**DOI:** 10.1128/MRA.00547-19

**Published:** 2019-08-15

**Authors:** Hiroto Ide, Kento Ishii, Hirotsugu Fujitani, Satoshi Tsuneda

**Affiliations:** a Department of Life Science and Medical Bioscience, Waseda University, Tokyo, Japan; b Biomedical Research Institute, National Institute of Advanced Industrial Science and Technology (AIST), Tsukuba, Ibaraki, Japan; c Research Organization for Nano and Life Innovation, Waseda University, Tokyo, Japan; Georgia Institute of Technology

## Abstract

Here, we report the draft genome sequence of Acidovorax sp. strain NB1, isolated from an enrichment culture of nitrite-oxidizing bacteria (NOB). Genes involved in denitrification were found in the draft genome of NB1. The closest strain to NB1 based on genomic relatedness is Acidovorax sp. strain GW101-3H11, with 91.5% average nucleotide identity.

## ANNOUNCEMENT

Nitrogen exists in various forms, and microorganisms mediate its circulation in natural environments and engineered systems. Ammonia (NH_3_) is eventually converted to nitrogen gas (N_2_) through nitrification and denitrification, which are the key processes of the global nitrogen cycle. Acidovorax spp., which are of the family *Comamonadaceae* in the order *Betaproteobacteriales,* are known as denitrifying bacteria and contribute to biological nitrogen removal in wastewater treatment plants (1–3).

Here, we announce the draft genome sequence of Acidovorax sp. strain NB1, isolated from a nitrite-oxidizing bacterium (NOB) enrichment culture, and the genes involved in denitrification. Previously, our group enriched the environmentally important NOB from the genus “*Candidatus* Nitrotoga” in mineral salts medium, in which they likely provided by-products and nitrate to heterotrophic bacteria (4). Among the heterotrophs, *Acidovorax* sp. NB1 was isolated from the “*Candidatus* Nitrotoga” enrichment culture using a nutrient broth (NB) medium gelled by agar.

The pure culture of NB1 was cultivated in the NB medium at 29°C prior to DNA extraction and genome sequencing. The NB1 genome was sequenced with an Illumina MiSeq instrument and QIAseq FX DNA library kit, which generated paired-end read sequences of 301 bp at 263.5× coverage, on average. Platanus_trim (http://platanus.bio.titech.ac.jp/pltanus_trim) was used to remove adapters and low-quality reads. The draft genome sequence of NB1 was assembled using Platanus v1.2.4 (5) and comprised 60 contigs with a total genome size of 5,493,953 bp (65.3% G+C content; *N*_50_, 332.59 kbp). Automated annotation of the coding sequence (CDS) region was performed using DFAST (6), generating 5,009 CDS features, 50 tRNAs, and 6 rRNAs. The genome sequences assigned to the genus *Acidovorax* were downloaded using NCBI genome downloading scripts (https://github.com/kblin/ncbi-genome-download), and the average nucleotide identity (ANI) was calculated using the Python module PYANI v0.2.8 (7). The ANI values between NB1 and the sequenced *Acidovorax* genomes were 81.9 to 91.5%. All software was run with default settings.

**FIG 1:**
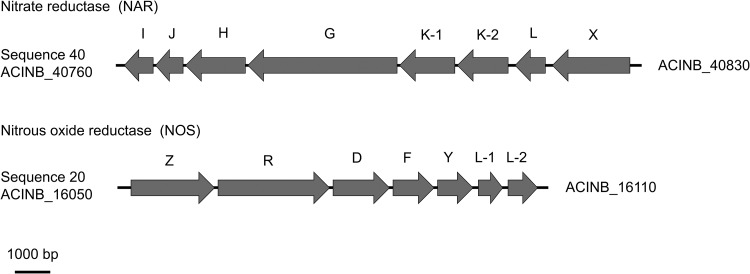
Schematic representation of the nitrate reductase (NAR) and nitrous oxide reductase (NOS) operons of *Acidovorax* sp. NB1. Each arrow represents a CDS with the gene subunit letter identity indicated above the arrow.

NB1 has the whole gene set necessary for nitrate reduction (*narK2K1GHJI*), and the gene that regulates their transcription, *narXL,* is located upstream (Fig. 1). The *narXL*-*narK*-*narGHJI* operon is conserved in most *Betaproteobacteriales* genomes (8, 9). Dissimilatory-nitrite-reduction-related genes, such as *nirK* or *nirS*, were not found in the NB1 genome. Other *Acidovorax* strains that have nitrate reduction potential lack dissimilatory nitrite reductases (10–12). The *nosZRDFYLL* operon involved in nitrous oxide (N_2_O) reduction is present in the NB1 genome, and *nosR*, the regulator gene of *nosZ*, lies downstream of its cluster. The order of nitrous oxide reductases is the same as that of “*Candidatus* Magnetobacterium” spp. of the phylum *Nitrospirae* (13). With respect to nitric oxide (NO) reduction, only a nitric oxide reductase large subunit gene (*norB*) was identified in the NB1 genome. Considering that NB1 has a nitrate reductase gene cluster, reciprocal feeding may occur between NB1 and “*Candidatus* Nitrotoga” spp. in the enrichment culture.

### Data availability.

The draft genome sequence of *Acidovorax* sp. NB1 has been deposited in DDBJ/ENA/GenBank under the accession number BJHU00000000. The BioProject number is PRJDB5517, and the BioSample number is SAMD00074225. The associated raw sequence data have been deposited in the NCBI under the accession number DRP005091.
